# Assessing the Reliability of a Combat Sports Kick-Time Device

**DOI:** 10.3390/s25051420

**Published:** 2025-02-26

**Authors:** Johan Robalino, Ana Luiza Costa e Silva Cabral, Emerson Franchini, Márcio Fagundes Goethel, João Paulo Vilas-Boas, Bruno Mezêncio, Jacielle Carolina Ferreira

**Affiliations:** 1 Postgraduate Program in Physical Education, Federal University of Mato Grosso, Cuiabá 78060-900, Brazil; up202310701@up.pt (J.R.); jacielle.ferreira@ufmt.br (J.C.F.); 2 Centre for Research, Education, Innovation and Intervention in Sport (CIFI2D), Faculty of Sports, University of Porto, 4200-450 Porto, Portugal; jpvb@fade.up.pt; 3 Porto Biomechanics Laboratory (LABIOMEP), University of Porto, 4200-450 Porto, Portugal; up202203218@up.pt; 4 Martial Arts and Combat Sports Research Group, Sport Department, School of Physical Education and Sport, University of São Paulo, São Paulo 05508-060, Brazil; efranchini@usp.br; 5 Biomechanics Laboratory, School of Physical Education and Sport, University of São Paulo, São Paulo 05508-060, Brazil; mezencio@usp.br

**Keywords:** performance assessment, contact mat, sports technology, roundhouse kick, Arduino, biomechanics, karate

## Abstract

In combat sports, precise technique evaluation is crucial for performance optimization; however, traditional systems for evaluating kick performance are frequently unreasonably complicated and costly. This study offers a useful and accessible substitute by introducing a contact mat-based tool that measures the roundhouse kick’s execution time during both the attack and recovery phases and by demonstrating its reliability. The experimental sessions involved 16 male Shotokan karate athletes (age: 25.6 ± 7.1 years; height: 1.74 ± 0.05 m; body mass: 71.5 ± 8.7 kg; body fat percentage: 14.7 ± 6.7%; training experience: 11.0 ± 4.9 years). The protocol included four sessions, starting with a familiarization phase followed by three testing sessions (test, retest, and retest two), during which a standardized warm-up was performed along with the roundhouse kick test. The intraclass coefficient correlation (ICC) used indicated high reliability for the at-tack (ICC = 0.85, 95% CI [0.64, 0.94]), recovery (ICC = 0.89, 95% CI [0.75, 0.96]), and total time (ICC = 0.90, 95% CI [0.76, 0.96]). The Friedman test revealed no significant difference between testing sessions (*p* > 0.31), demonstrating high reliability and no significant differences between sessions. This study confirms the system as a simple and reliability tool for measuring roundhouse-kick timing in combat sports.

## 1. Introduction

Combat sports are characterized by their intense physical engagement, where the winner is determined by specific criteria established by the rules of each sporting modality [1,2]. These sports can be classified into three main categories: grappling, striking, and mixed combat sports. Grappling disciplines include Brazilian jiu-jitsu, judo, and wrestling, which focus on throwing techniques and ground combat through techniques such as immobilization, chokeholds, and joint locks [3]. Conversely, striking sports, including boxing, karate, kickboxing, taekwondo, and Muay Thai, emphasize the application of offensive techniques such as punches and kicks. These techniques must be executed in the shortest possible time and require the application of strength with high impulse for effective execution [1,2]. Additionally, there are mixed combat sports, such as hapkido and mixed martial arts, which combine techniques for both striking and grappling combat sports [4].

Among the common techniques in striking sports is the roundhouse kick, known by various names depending on the discipline: *mawashi geri* in karate [5], *bandal tchagui* in taekwondo [6,7,8], *tei chiyang* in Muay Thai [9], and round kick in kickboxing [10]. The roundhouse kick is performed through hip rotation, followed by hip flexion and rapid knee extension, propelling the leg in a circular arc and making impact with the instep or, in some variations, with the shin [11] According to Corcoran et al. [12], the speed of the kick is influenced by several factors categorized into four main domains: technical proficiency, lower body strength and flexibility, effective mass, and target-specific characteristics. Scientific evidence indicates that practitioners with higher technical proficiency can achieve greater speeds in kick execution [11,13,14].

Execution speed is a critical determinant of competitive success in combat sports, as it directly influences scoring opportunities [15]. For instance, research by Estevan et al. [7] demonstrated that medal-winning taekwondo athletes achieved significantly shorter attack-phase times in roundhouse kicks to the head compared to non-medalists, both at short and medium distances. These findings underscore that faster execution not only improves effectiveness but also constitutes a decisive factor in high-level performance. Since technical effectiveness heavily relies on proper execution, precise monitoring becomes highly recommended in these sports [1,2]. Such monitoring enables coaches and athletes to identify and correct errors, optimize performance, and support ongoing technical refinement [16]. Additionally, motion analysis provides precise feedback, which is instrumental in advancing skill acquisition and enhancing competitive readiness [17].

The assessment of kicks in combat sports is typically conducted by measuring foot speed in a laboratory setting using camera-based motion capture systems, regarded as the gold standard for kinematic analysis [9,11,13,14]. However, these analyses require a highly controlled environment, which does not always reflect the actual conditions of combat. Furthermore, these systems are costly and necessitate specialized equipment, both in terms of hardware (high-speed cameras, amplifiers, markers, etc.) and software for data analysis [5]. The use of markers on specific body points can be uncomfortable for the athlete and potentially alter their natural performance [18]. Finally, the setup of the equipment and the processing of the obtained data are time-consuming tasks, making them impractical in situations that require real-time evaluation [19]. All these aspects can justify the scarcity of quantitative evaluation of techniques such as the roundhouse kick.

In response to these limitations, various systems have been developed that combine force transducers with contact mats to measure impact force and execution time of different combat techniques [6,8]. However, these systems also present coupling limitations, as they must be mounted on a rigid structure to avoid precision loss due to the inertia generated by strikes [13]. On the other hand, the use of contact mats, which consist of two conductive surfaces that close an electrical circuit under minimal pressure (switch principle) [20], offers a viable alternative. Studies conducted by Tenelsen et al. [21] have demonstrated the reliability of contact mats for timing measurements, showing high test–retest concordance for evaluating drop jumps at various heights (ICC: 0.70–0.92; SEM: 8.5–18.4 ms; CV: 3.6–6.4%). Given their practicality and affordability, a system based on contact mats could provide a more accessible and portable solution for measuring kick execution time in combat sports. This would allow real-time evaluation in environments that more closely resemble the conditions in which athletes are accustomed to train. This would allow for a more accessible and less invasive analysis of athlete performance, which is particularly beneficial for coaches and athletes interested in improving the technique and effectiveness of their strikes without the need for costly laboratory work, offering a more affordable alternative compared to traditional laboratory setups.

Thus, the purpose of this study was to describe the assembly of a device based on contact mats that allows for the measurement of execution time of the roundhouse kick in karate athletes and to demonstrate its reliability (including both the attack and return phases), which is essential for coaches and researchers who need accurate and accessible tools. Our hypothesis was that the measurement procedure for the temporal variables used to evaluate the roundhouse kick in karate athletes would exhibit a high degree of reliability, which would be reflected in the consistency of results obtained across multiple testing sessions with the same individuals.

## 2. Materials and Methods

### 2.1. Kick-Time Device

**Figure 1:**
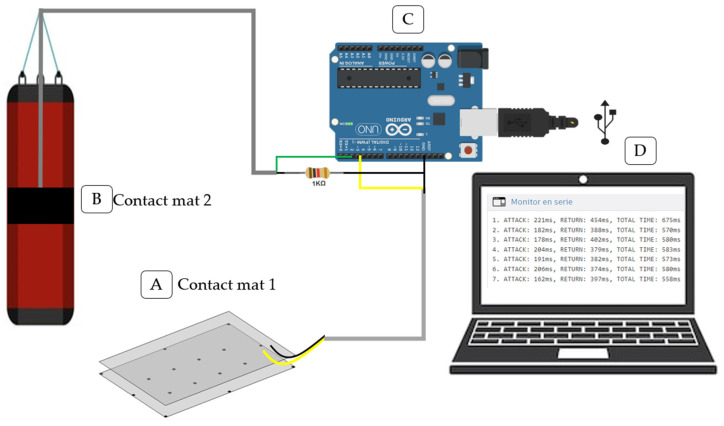
Kick time device configuration: (**A**) contact mat 1, (**B**) contact mat 2 on punching bag, (**C**) Arduino Uno R3 microcontroller, (**D**) computer with Arduino IDE 1.8.19 for Windows.

The kick-time device includes two contact mats: one positioned on the ground (Figure 1, detail A) for the supporting foot and another embedded within the striking bag (Figure 1, detail B). These mats are connected through an Atmega328 microcontroller (Microchip Technology, Chandler, AZ, USA) mounted on an Arduino Uno board (Arduino LLC, Monza, Italy), shown in Figure 1, detail C, which is linked to a computer via USB serial communication to interface with the Arduino IDE 1.8.19 (Arduino LLC, Italy), as shown in Figure 1, detail D. This setup allows real-time data visualization through the serial monitor, as shown in Figure 1.

Each contact mat consisted of two layers of flexible fabric and two conductive steel sheets (50 × 60 cm, 2–3 mm thick) functioning as pressure sensors [22]. The fabric layers were precisely cut to match the dimensions of the conductive sheets, ensuring alignment. Between the conductive sheets, 3 mm thick EVA (ethylene-vinyl acetate) rubber spacers were placed to maintain a gap when no pressure was applied, enabling contact only upon the application of force. The second conductive sheet was positioned atop the spacers, ensuring precise alignment with the first. Each sheet was connected to a dedicated wire, enabling the circuit to remain open when the sheets were separated (i.e., no pressure was applied) and to close when the sheets made contact due to an applied force, as illustrated in Figure 1, detail A. The first contact mat was placed on the ground at a self-selected distance by the athletes, while the second mat was embedded within the striking bag and adjusted to match the thickness of the conductive sheet. The cable from the ground mat was connected to pin 3 and the bag mat’s cable to pin 2 on the Arduino board.

The development of these contact mats was carried out by the research team, whose expertise encompasses electronics, sensor integration, and hardware design for data acquisition systems. Their experience in creating electronic devices tailored for biomechanical and sports research applications ensured that the mats met the technical and experimental requirements of the study.

An interrupt-based code was implemented to measure the time intervals between events on the microcontroller platform (Appendix A). This system employs two interrupt pins configured to detect changes in the conductive material’s state, triggering specific functions for time recording and interval calculation. The function associated with the ground mat manages events during the execution of the kick. When the athlete’s foot loses contact with this mat, a timer is initiated until contact is established with the second mat within the striking bag. Upon impact, the circuit closes, halting the timer, which records this time as the attack time.

Following impact, as the foot disengages from the bag, the circuit reopens, starting a new count, which stops when the athlete’s foot touches the ground mat. The circuit’s closure at this stage determines the return time. The sum of the attack and return times yields the total kick time. The system utilizes global variables for time management and interrupt logic control, using the serial port for immediate data display. This approach enables automatic and precise time interval measurement, eliminating the need to continuously use the main loop for event detection.

The system’s performance parameters were defined through code specifications under controlled laboratory conditions. The minimum detectable time was set at 100 ms, determined by the function minElapsed in the code and influenced by the 1 ms resolution of the millis () function, along with a stabilization delay. The estimated standard error of the system was approximately 1 ms, attributed to the inherent precision of the millis () function and potential latency during interrupt handling and sensor activation. These parameters ensure the system’s reliability for accurate detection of activation times within the scope of this research.

### 2.2. Experimental Procedures

This study included four experimental sessions, each conducted on separate days with 48 h intervals to ensure recovery between trials. In each session, participants followed a standardized warm-up protocol, starting with a 10 min stationary cycling routine at a self-selected, comfortable intensity, followed by five submaximal kicks to prepare for the *mawashi geri* test.

During the initial session or familiarization, participants were oriented to the experimental procedures, provided informed consent, and underwent anthropometric assessments. Measurements included body mass, recorded using a Multilaser HC021 scale (Multilaser, São Paulo, Brazil; precision: 0.1 kg); height, measured with an Avanutri Stadiometer (Avanutri, Rio de Janeiro, Brazil; precision: 0.1 cm); and body fat percentage, estimated with a Harpenden C-136 Analog Caliper (Harpenden, UK; precision: 0.1 mm) following the Jackson and Pollock [23] seven-skinfold protocol. The height of the measurement devices was adjusted to match an average kick height, and this setting was recorded to ensure consistency across subsequent sessions. To familiarize athletes with the protocol, participants then performed three roundhouse kick attempts on the kick timing device.

**Figure 2:**
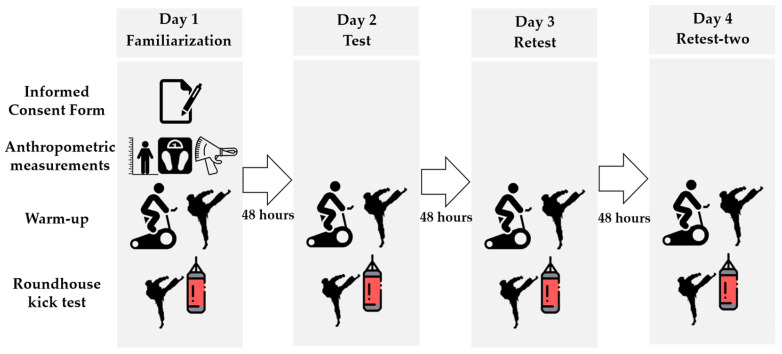
Experimental procedures.

In the second (test), third (retest), and fourth (retest two) sessions, both the warm-up and roundhouse kick procedures were repeated exactly as in the initial session, as shown in Figure 2.

### 2.3. Participants

Using G*Power software (version 3.1.9.4; Düsseldorf University, Düsseldorf, Germany), the minimum sample size for this study was calculated to ensure statistical power. An a priori analysis with ANOVA for repeated measures, focusing on execution time for medium-distance kicks, used parameters from Estevan et al. [7]: power (1-β) = 0.80, effect size (f) = 0.86, α = 0.05, and a correlation of 0.5. The analysis determined that 16 participants were required to reliably detect differences in execution time.

Consequently, 16 male Shotokan karate competitors at the national level were included in the participant group, classified as highly trained/national level according to [24]. The group consisted of two brown belts and fourteen black belts, with the following characteristics: age: 25.6 ± 7.1 years; height: 1.74 ± 0.05 m; body mass: 71.5 ± 8.7 kg; body fat percentage: 14.7 ± 6.7%; training experience: 11.0 ± 4.9 years. Participants were required to have participated in at least the most recent national championships and to have trained in strength and karate-specific activities for a minimum of one year. They attended tactical, technical, and physical training sessions at least three times a week. Additionally, participants needed to be free of musculoskeletal injuries for six months prior to the study. With no scheduled competitions for the remainder of the year, all athletes were in a training transitional phase. Written informed consent was obtained from all participants after they were fully briefed on the study’s objectives. The Federal University of Mato Grosso (UFMT) Research Ethics Committee approved all procedures under CAAE number 33016620.3.0000.8124. This study adhered to the principles of the Helsinki Declaration throughout.

### 2.4. Roundhouse Kick Test

According to Wąsik et al. [11], the roundhouse kick is categorized as a swing-type kick because it begins with a hip flexion phase and then quickly extends the knee until it makes contact with the instep. Using a kick timing device, athletes executed the roundhouse kick with a self-selected distance and their preferred leg, emulating the intensity of actual combat. The attacking foot was positioned behind and in line with the first contact mat to start the process. The kick was performed using standard karate technique after a brief period of mental preparation. For the kick to be considered valid, the attacking foot had to strike the second contact mat within the designated area for a middle kick using the instep, and then land back on the first mat. Additionally, the movement had to follow the correct form of a roundhouse kick. One evaluator, a second dan black belt with twenty years of karate experience, assessed each attempt’s validity. Three legitimate attempts were required by the test protocol, with a one-minute break in between to ensure adequate recovery and minimize fatigue effects. The mean value over the three attempts was computed for statistical analysis.

### 2.5. Statistical Analysis

Data normality was assessed using the Shapiro–Wilk test. The data demonstrated a non-normal distribution, imposing the use of non-parametric statistical tests for analysis. Data are presented as the median (Med) and interquartile range (IQR). To identify differences between the test, retest, and retest-two measurements of attack time, return time, and total kick time, paired Friedman tests were conducted. Alongside these analyses, we computed the intraclass correlation coefficient (ICC) using a two-way mixed-effects model for absolute agreement, ICC(2,1). This metric, ranging from 0 (no agreement) to 1 (perfect agreement), was calculated as ICC = (MS_B_ − MS_W_)/[MS_B_ + (k − 1) MS_W_], where MS_B_ is the mean square between subjects, MS_W_ is the mean square within subjects, and k is the number of measurements per subject [25]; the coefficient of variation (CV) to assess data dispersion, defined as CV = (SD/Mean) × 100, where SD is the standard deviation and mean is the average of the measurements; and Percentage Standard Error of Measurement (%SEM) was calculated to assess random variation, using the formula SEM = SD × √(1 − r) and 95% confidence intervals (CI), as in the case of ICC and Bland–Altman. According to Bradshaw et al. [26], the following cut-off points were established: an ICC less than 0.67 and a CV greater than 10% indicate high variability (i.e., low reliability); moderate variability occurs if only one of these criteria is met; and low variability (i.e., good reliability) is indicated by an ICC greater than 0.67 [27]. The presentation of results was facilitated through the utilization of the Bland–Altman method, with a predefined agreement level of 95% [28]. All analyses were conducted using IBM SPSS Statistics 27 software (IBM, Armonk, NY, USA). Statistical significance was set at *p* ≤ 0.05.

## 3. Results

Reliability results are presented in Table 1, indicating no significant differences among the test, retest, and retest-two comparisons.

**Figure 3:**
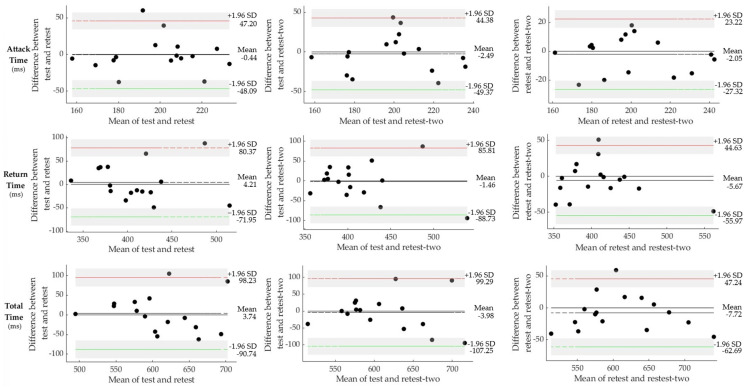
Bland–Altman plot representing the agreement of mean values for attack time, return time, and total time between test, retest, and second retest, within a 95% confidence interval. ms: milliseconds.

The Bland–Altman plot in Figure 3 illustrates the mean values for attack time, return time, and total time across the test, retest, and second retest, demonstrating agreement within a 95% confidence interval. For the variable attack time, 93.75% of the samples were within the confidence intervals between the test and retest, 100% between the test and second retest, and 100% between the retest and second retest. For the variable return time, 93.75% of the samples were within the confidence intervals between the test and retest, 100% between the test and second retest, and 100% between the retest and second retest. For the variable total time, 100% of the samples were within the confidence intervals between the test and retest, 93.75% between the test and second retest, and 100% between the retest and second retest.

## 4. Discussion

This study aimed to develop a contact mat-based device for measuring the execution time of the roundhouse kick and to assess its reliability. It was hypothesized that the device would demonstrate a high degree of reliability in measuring time-related variables associated with this technique. The results confirmed this hypothesis, as repeated trials with the same participants showed remarkable consistency across sessions, with no significant differences in the measurements of attack and return times, reflecting the high reliability of the device.

Temporal analysis of attack techniques is essential for the development of competitive strategies, as it allows for a deeper understanding of the patterns and tactics used by athletes in the practice of combat sports. The most widely used method and considered the gold standard for performing these analyses in combat sports is high-frequency motion capture [13,29,30]. The study by Pozo et al. [30], for example, obtained execution times for the front kick using motion capture and force platforms and reported coefficients of variation of 2.9% (1.2) in international athletes and 6.1% (2.5) in national athletes. Our device demonstrated comparable reliability in measuring these temporal variables. Therefore, unlike widely used technologies that are costly and whose complexity restricts their use outside of the laboratory, our study demonstrated that inexpensive and portable devices could be used in the practice of combat sports without compromising precision.

The successful execution of the roundhouse kick requires athletes to achieve high movement velocities, demonstrating its adaptability as a technique [13,14]. A previous study using five piezoelectric sensors in a pentagonal inertially relevant structure on a dummy showed that medal-winning athletes tended to record shorter attack times than non-medal-winning athletes (248 ± 12 ms vs. 295 ± 13 ms, respectively; *p* < 0.01) during this technique [7]. In our study, the average times recorded were 205 ms (IQR 76), 201 ms (IQR 79), and 193 ms (IQR 83), suggesting that participants achieved competitive levels. While these differences may not be easily discernible to coaches, especially at elite levels, the results emphasize the importance of precise tools for performance evaluation, as shorter attack times are often associated with higher technical skill and better physical preparation. Therefore, having an assessment tool that is practical and easily applicable in the daily practice of these athletes can help improve performance in competitions.

Regarding the return phase, our study showed average times of 602 ms (IQR 249), 608 ms (IQR 225) and 588 ms (IQR 229). The speed of return of the foot after contact is crucial to prepare offensive or defensive maneuvers, but this phase is often underestimated in the literature, raising important questions about its impact on technical performance [31]. The studies by Piemontez et al. [32] reported times much higher (810 ms and 980 ms, respectively) than the times reported in this study, despite having used a similar sample (male black belt karate athletes). Therefore, this variability can be explained by the evaluation methods and the instructions provided regarding the return. This highlights the need to standardize the evaluation procedures of the techniques used by athletes in daily practice, employing instruments that are accessible and applicable to the context of combat sports.

In our previous study, Robalino et al. [33], we employed an S-type load cell system combined with a contact mat on a structure designed to minimize inertial interference to evaluate temporal variables in combat sports. This approach demonstrated reliability in test–retest sessions for attack time (ICC = 0.70; CV = 0.14%; SEM = 8.06), return time (ICC = 0.71; CV = 4.28%; SEM = 12.20), and total time (ICC = 0.77; CV = 2.88%; SEM = 8.49%), aligning with the findings of the present study for the attack phase. However, the return phase and total time exhibited moderate variability. For the three variables analyzed, ICC exceeded 0.70, reflecting good relative reliability. Absolute reliability, as assessed by the CV, also showed favorable values; however, the SEM was relatively high. This suggests that, although the measurements demonstrate acceptable reliability, potential sources of error, particularly for longer durations such as the return phase and total time, impose further investigation to improve precision. A plausible explanation for these discrepancies is that, unlike the controlled conditions of our protocol, actual combat scenarios do not constrain athletes to return the striking limb to its original position, introducing variability that may influence these temporal parameters.

Although the use of a two-contact mat offers significant advantages in combat sports practice, certain limitations must be acknowledged. A gold standard validation could not be performed, making it impossible to compare results with other devices. The device cannot measure impact force or analyze detailed kinematic variables. Moreover, the lack of stable mat fixation introduces variability due to bag movement after impact, rendering the measurement of contact time unfeasible. Despite these constraints, the device provides a cost-effective alternative for evaluating technical performance in combat sports. Its capacity for real-time measurements facilitates targeted training adjustments and the optimization of competitive strategies. Furthermore, its portable design and affordable price make it a practical tool for coaches and athletes to track and monitor technical performance for athletes in real-life situations.

## 5. Conclusions

The findings indicate that the procedure demonstrates high reliability in measuring timing variables related to attack time, return time, and total kick time. This methodology, which integrates contact mats synchronized with a microcontroller on an inertially relevant structure like a punching bag, offers a practical and affordable alternative to the evaluation methods considered too costly, such as motion capture systems and other traditional methods that require complex equipment and controlled laboratory conditions.

## Figures and Tables

**Table 1:** Reliability analysis of kinematic variables for the roundhouse kick.

Variables	Test Med (IQR)	Retest Med (IQR)	Retest Two Med (IQR)	*p* Value	ICC (95% IC)	CV%	SEM%
Attack Time (ms)	205 (76)	201 (79)	193 (83)	0.305	0.85 (0.64, 0.94)	0.80	2.94
Return Time (ms)	400 (190)	414 (204)	397 (224)	0.779	0.89 (0.75, 0.96)	0.96	22.26
Total Time (ms)	602 (249)	608 (225)	588 (229)	0.646	0.90 (0.76, 0.96)	0.91	18.05

Med: median; IQR: interquartile range; *p* = probability value; ICC: intraclass correlation coefficient; CV%: coefficient of variation; SEM%: Standard Error of Measurement; ms: milliseconds.

## Data Availability

The datasets analyzed during the current study are available from the corresponding author.

## Appendix A

**1**	**const byte interruptPin_1 = 2;**
**2**	**const byte interruptPin_2 = 3;**
**3**	
**4**	**volatile *unsigned long* minElapsed = 100L;**
**5**	**volatile *unsigned long* elapsedTime;**
**6**	**volatile *unsigned long* previousTime = millis();**
**7**	
**8**	***unsigned long* t0 = 0L;**
**9**	***unsigned long* t1 = 0L;**
**10**	
**11**	***int* TA;**
**12**	***int* TR;**
**13**	
**14**	
**15**	***bool* teste = false;**
**16**	
**17**	***void* setup() {**
**18**	**Serial.begin(115200);**
**19**	
**20**	**pinMode(interruptPin_1, INPUT_PULLUP);**
**21**	**pinMode(interruptPin_2, INPUT_PULLUP);**
**22**	**attachInterrupt(digitalPinToInterrupt(interruptPin_1),tapete,CHANGE);**
**23**	**attachInterrupt(digitalPinToInterrupt(interruptPin_2),saco,CHANGE);**
**24**	
**25**	**}**
**26**	
**27**	***void* loop(){**
**28**	**if (teste){**
**29**	**Serial.print(TA);**
**30**	**Serial.print(“;”);**
**31**	**Serial.println(TR);**
**32**	**teste = false;**
**33**	**}**
**34**	**}**
**35**	
**36**	***void* tapete(){**
**37**	**elapsedTime = millis() − previousTime;**
**38**	**if (elapsedTime > minElapsed){ //true interrupt**
**39**	**previousTime = millis();**
**40**	**delay(5);**
**41**	**if (digitalRead(interruptPin_1)){**
**42**	**if (t0){**
**43**	**Serial.println(“Contato Tapete—Fim”);**
**44**	**TA = t1 − t0;**
**45**	**TR = previousTime − t1;**
**46**	**teste = true;**
**47**	**t0 = 0L;**
**48**	**t1 = 0L;**
**49**	}
**50**	**else {**
**51**	**Serial.println(“Contato Tapete—Inicio”);**
**52**	**t0 = 0L;**
**53**	**t1 = 0L;**
**54**	**}**
**55**	**}**
**56**	**else {**
**57**	**if (!t0){**
**58**	**Serial.println(“Saida Tapete—T0”);**
**59**	**t0 = previousTime;**
**60**	**}**
**61**	**}**
**62**	**}**
**63**	**}**
**64**	
**65**	***void* saco(){**
**66**	**elapsedTime = millis() − previousTime;**
**67**	**if (elapsedTime > minElapsed){ //true interrupt**
**68**	**previousTime = millis();**
**69**	**delay(5);**
**70**	**if (digitalRead(interruptPin_2)){**
**71**	**if (t0){**
**72**	**Serial.println(“Contato Saco—T1”);**
**73**	**t1 = previousTime;**
**74**	**}**
**75**	**}**
**76**	**}**
**77**	}
